# Demographic and ecogeographic factors limit wild grapevine spread at the southern edge of its distribution range

**DOI:** 10.1002/ece3.7519

**Published:** 2021-05-08

**Authors:** Oshrit Rahimi, Noa Ohana‐Levi, Hodaya Brauner, Nimrod Inbar, Sariel Hübner, Elyashiv Drori

**Affiliations:** ^1^ Department of Chemical Engineering Ariel University Ariel Israel; ^2^ Independent Researcher Ashalim Israel; ^3^ The Samson Family Grape and Wine Research Center Eastern Regional R&D Center Ariel Israel; ^4^ Department of Civil Engineering Ariel University Ariel Israel; ^5^ The Department of Geophysics and Space Science Eastern Regional R&D Center Ariel Israel; ^6^ Galilee Research Institute (Migal) Tel‐Hai Academic College Upper Galilee Israel

**Keywords:** ecogeographic constraints, maxent, multivariate spatial clustering, phenotypic diversity, population genetic structure, wild grapevine

## Abstract

The spatial distribution of plants is constrained by demographic and ecogeographic factors that determine the range and abundance of the species. Wild grapevine (*Vitis vinifera* ssp. *sylvestris*) is distributed from Switzerland in the north to Israel in the south. However, little is known about the ecogeographic constraints of this species and its genetic and phenotypic characteristics, especially at the southern edge of its distribution range in the Levant region. In this study, we explore the population structure of southern Levantine wild grapevines and the correlation between demographic and ecogeographic characteristics. Based on our genetic analysis, the wild grapevine populations in this region can be divided into two major subgroups in accordance with a multivariate spatial and ecogeographical clustering model. The identified subpopulations also differ in morphological traits, mainly leaf hairiness which may imply adaptation to environmental stress. The findings suggest that the Upper Jordan River population was spread to the Sea of Galilee area and that a third smaller subpopulation at the south of the Golan Heights may represent a distinguished gene pool or a recent establishment of a new population. A spatial distribution model indicated that distance to water sources, Normalized difference vegetation index, and precipitation are the main environmental factors constraining *V. v*. *sylvestris* distribution at its southern distribution range. These factors in addition to limited gene flow between populations prevent further spread of wild grapevines southwards to semi‐arid regions.

## Graphical abstract

In this paper, genetic analysis, spatial, and eco‐geographical clustering models show there are two major subpopulations of wild grapevine in the south Levant, differing morphologically mainly in leaf hairiness. We also showed that closeness to water sources, medium‐high NDVI levels, and sufficient precipitation rates are the main effectors possibly preventing further distribution of wild grapevines southwards.

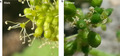

## 1 INTRODUCTION

Wild grapevine, *Vitis vinifera* L. subsp. *sylvestris* (Gmelin) Hegi (hereafter *V. v*. *sylvestris*), is considered the ancestor of the domesticated *Vitis vinifera* L. subsp. *vinifera* (*V. v*. *vinifera*) (Hegi, 1925; Heywood & Zohary, 1995; Zohary & Spiegel‐Roy, 1975). Thus, preservation of its germplasm may prove important for future breeding programs (Duchêne et al., 2012). Yet, recently accelerated development and urbanization processes have dramatically reduced the habitats of *V. v*. *sylvestris* and severely damaged natural populations. Today, the wild grapevine is considered an endangered species of high priority for conservation (Biagini et al., 2014; Ocete, Arroyo‐García, et al., 2011).

Wild grapevine is believed to have originated in the Caucasian region (Grassi et al., 2006; Heywood & Zohary, 1995) and disperse over long distances mainly by birds, throughout its distribution range (Hegi, 1925) from Western Europe to Eastern and Central Asia (Arroyo‐García & Revilla, 2013). *V. v*. *sylvestris* is a dioecious species where the male flower contains erect stamens and does not contain female organs, while the female flower contains an ovary, stylus, and degenerated stamens (Spada et al., 2003; Zohary & Spiegel‐Roy, 1975). The fruits develop from the female flower and are usually characterized by a thin bunch of round, small dark berries, and an oval pit (Ocete et al., 2011). Until the mid‐19th century, the wild grapevine was abundant across Europe; however, its distribution dramatically decreased with the penetration of pathogens (e.g., phylloxera, powdery, and downy mildews) from North America (This et al., 2006).

Recent studies examined the genetic diversity and the spatial distribution patterns of wild grapevines using primarily simple sequence repeat (SSR) markers (Biagini et al., 2014; De Andrés et al., 2012; Ergül et al., 2011; Schneider et al., 2015; Zoghlami et al., 2013). For example, a study conducted across different regions in Spain showed that *V. v*. *sylvestris* populations grow in a wide range of habitats including sheers and beaches, forests, and riverbeds (De Andrés et al., 2012). Following an extended survey that included recording of ecological and topographic traits of *V. v*. *sylvestris* in Italy, it was shown that habitats in altitudes below 300 m with access to water sources, high vegetation density, low anthropogenic disturbance, and potential correlation with carbonatic soil substratum are usually more favorable for establishment of stable populations (Biagini et al., 2014). The distribution of wild grapevine is also sensitive to biotic factors including mites, powdery mildews, and downy mildews (De Andrés et al., 2012).

**FIGURE 1:**
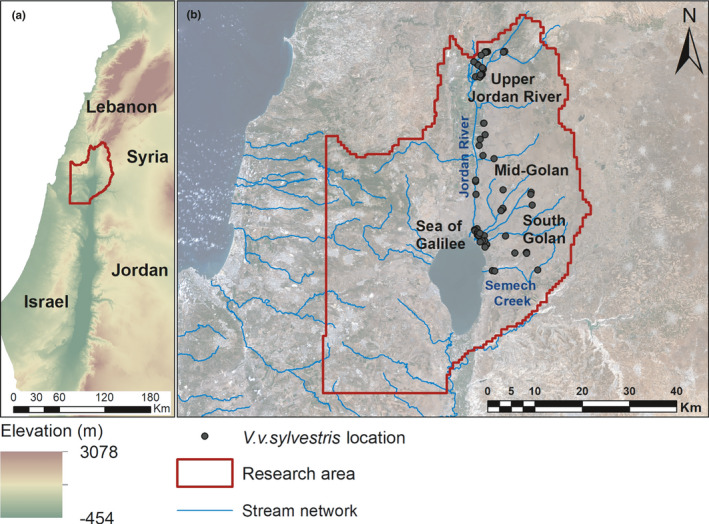
Distribution of *V. v*. *sylvestris* in the Southern Levant. (a) The study area of the wild grapevine collection in the northern part of Israel. (b) The study area, with sampled accessions marked by black dots and streams and river networks marked with blue lines

An early study on the distribution of grapevines in the old world, including the southern Levant (where today Israel, southern Syria, and southern Lebanon are present, Figure 1a), has failed to identify wild grapevine populations in Israel (Zohary & Spiegel‐Roy, 1975). A decade later, a population of *V. v*. *sylvestris* was identified on the Jordan River banks at the Upper Galilee region and marked the southern distribution edge of wild grapevine (Rottenberg, 1998). More recently, a comprehensive survey conducted throughout Israel was able to locate additional populations in the Upper Galilee region and around the Sea of Galilee (Drori et al., 2015, 2017; Drori, Rahimi, et al., 2017). Some of the collected wild grapevines were sequenced using next‐generation sequencing (NGS) techniques, and their single nucleotide polymorphism (SNP) data were initially compared to those of domesticated endogenous varieties (Drori et al., 2017; Drori, Rahimi, et al., 2017). However, none of these studies systematically explored the ecology, nor the genetic or phenotypic characteristics of wild grapevines along the southern edge of the species distribution range.

Species distribution range is dictated by evolutionary and ecological processes (Bridle & Vines, 2007; Sexton et al., 2009). Environmental factors could be both abiotic and biotic (Leach et al., 2016; Lewis et al., 2017; Murcia, 1995) demographic factors include genetic isolation (Sexton et al., 2009), absence of gene flow, high inbreeding, and low genetic variation. Adaptation of populations to new environments relies on standing genetic variation or accumulation of new mutations (Barrett & Schluter, 2008). Thus, genetic variation is an important factor for long‐term adaptive potential of a population (Bouzat, 2010).

In the past few decades, new powerful computational tools were developed to explore the spatial distribution patterns and associated diversity in natural populations. Among these tools are ecological niche models (ENM) and species distribution models (SDM) (Guisan & Thuiller, 2005; Wiens & Graham, 2005). A variety of algorithms for SDM are now available, including machine learning‐based approaches (Olden et al., 2008; Segurado & Araújo, 2004). One of the popular tools to study SDMs is a maximum entropy‐based (Maxent) niche modeling technique (Phillips et al., 2006), which allows to estimate the distribution range of a species and the main contributing environmental factors (Rhoden et al., 2017; Slater & Michael, 2012). Another widely applied approach to study the distribution of a species is a multivariate clustering (Hargrove & Hoffman, 1999), which considers many variables in order to partition the dataset into groups or clusters. A well‐clustered set of observations is the set of entities that shows higher similarity within the cluster than to entities in other clusters (Everitt, 1980). In the past decade, several such clustering approaches have been proposed to assess the presence of spatial autocorrelation in a dataset (Córdoba et al., 2013; Peeters et al., 2015) and have been widely applied in agriculture (Ohana‐Levi et al., 2019; Tardaguila et al., 2018), epidemiology (Mahara et al., 2016) and environmental modeling (Ren et al., 2016). However, only a few studies applied multivariate spatial clustering models to address species distribution problems (Feng et al., 2019; Feng et al., 2017).

In this study, we characterize the distribution constraints of *V. v*. *sylvestris* in the southern part of the Levant. We hypothesize that the wild grapevine distribution is limited by an interplay between demographic and environmental factors. To address this hypothesis, we use a wide set of tools to quantify the contribution of different parameters to the observed genetic and phenotypic variation among wild grapevine populations, at the southern distribution edge of the species.

## 2 MATERIALS AND METHODS

### 2.1 Plant material and research area

During the years 2012–2019, a comprehensive survey of wild grapevines was conducted (Drori, Levy, et al., 2017). A total of 129 *V. v*. *sylvestris* accessions were sampled in the Upper Galilee and around the Sea of Galilee in the northern part of Israel (Figure 1). Verification of the sylvestris identity of accessions and determination of their sex were performed based on their flower's structure: The male flower contains only stamens, and the female flower contains a full carpel and deteriorated stamens (Figure S1). For each plant, we sampled shoot tips or young leaves for DNA extraction. Sampling was conducted during the spring of each year, and samples were stored at −80°C until processing.

### 2.2 Multivariate spatial clustering

Seven continuous variables were selected based on previous studies of wild grapevine ecology and distribution and used in the spatial model (Biagini et al., 2014; Carey et al., 2002; Hunter & Bonnardot, 2011). The environmental data were prepared as raster grids using the ArcGIS software v10.6.1 (ESRI, 2018). All variables represented as grids were scaled to a spatial resolution of 30 m and included the following variables:

- Topography (slope and aspect): calculated from a digital elevation model (DEM) generated by the Survey of Israel (SOI). Slopes were calculated in degrees relative to a horizontal plane, and aspect was calculated as degrees relative to the North cardinal direction (0°).
- Distance to a water source: calculated from the streams layer generated by SOI, using the Euclidean distance tool in ArcGIS (ESRI, 2018).
- Normalized difference vegetation index (NDVI): generated for two dates (7 April 2019 and 12 July 2019) using Landsat 8 imagery. Two time points (summer and spring) were chosen because of the differences in vegetation density between these seasons; the spring period is characterized by higher vegetation compared to the dry summer period, when the vegetation remains mostly near water sources or at irrigated areas. The images were atmospherically corrected using the dark object subtraction method via the “RStoolbox” package in R (Leutner et al., 2019). The NDVI value was calculated as the ratio between the difference of reflectance from the near‐infrared (NIR) and red bands, and the sum of the reflectance and red bands, to provide a scale of the degree of photosynthetically active biomass (Tucker, 1979).
- Land surface temperature (LST): calculated based on the Landsat 8 thermal infrared (TIR) band (band 10) acquired on 12 July 2019 (Avdan & Jovanovska, 2016). The surface temperature was calculated using the “RStoolbox” (Leutner et al., 2019) and “raster” (Hijmans & Etten, 2012) packages in R.
- Precipitation: mean annual rainfall amounts (mm) based on a 30‐year average as measured by the Israel Meteorological Service.

The values of each of these variables were extracted for each of the accessions according to their geographic location, resulting in a total of 119 entities; each represents a single accession. To control for autocorrelation between variables, spatially varying attributes were clustered (Peeters et al., 2015). Using the 119 locations, the Getis‐Ord Gi* hot‐spot analysis (Getis & Ord, 1992) was performed for each variable, with a radius of 5,000 m using the R package “spdep” (Bivand & Wong, 2018). The Gi* hot‐spot analysis is a local method that relates to spatial subregions of the entities in space and is designed to quantify the degree of spatial autocorrelation among neighboring points. The output of this calculation is a *Z*‐score that is assigned to each point after standardization and indicates the degree of similarity between neighboring entities. *Z*‐score values far from zero signify strong spatial dependencies, and negative or positive values correspond to low or high values of the original variable, respectively (Luković et al., 2015). A complementary *p*‐value represents the significance level of the spatial relationship. A fuzzy c‐means (FCM) clustering algorithm was then applied with the Z‐score values of the different variables as input (Ohana‐Levi et al., 2020). A fuzzy set relies on the premise that boundaries between clusters (*C*) are not always discrete; thus, assignment of an observation to a specific cluster is not definite (Gath & Geva, 1989).

The output of the FCM algorithm includes a list of fuzzy membership values. Each observation receives a membership value between 0 and 1, denoting the degree of membership, or the probability of its placement to each cluster *C* (Akman et al., 2019). The observation is then assigned to a specific cluster according to the maximum fuzzy membership. The FCM algorithm was applied using the “ppclust” package in R (Cebeci, 2019). The number of *C* was determined by the admixed and correlated allele frequency models. Cluster separation was evaluated using the silhouette index (Rousseeuw, 1987). This index is based on the similarity of each object to other objects in their assigned cluster, compared to the dissimilarity of the objects to those of other clusters. The silhouette index was applied using the R package “clusterCrit” (Desgraupes, 2018). Finally, a McNemar's χ^2^ test (Hazra & Gogtay, 2016; McNemar, 1947) was performed to assess whether the clusters based on the genetic diversity analysis had a similar distribution to those generated by the multivariate spatial clustering method based on ecogeographic attributes.

### 2.3 Niche and distribution modeling analysis

To model the niche suitability and distribution of wild grapevine, a maximum entropy (Maxent) (Rhoden et al., 2017) analysis was conducted with the Maxent software (Phillips et al., 2020). The Maxent algorithm takes environmental information as a grid and georeferenced occurrence localities based on the sampling site locations and performs a suitability analysis across the grid. For the Maxent analysis, ten variables were used, seven of which were described in the multivariate spatial clustering section. In addition, the following categorical variables were included:

- Soil and lithology: set as categorical variables based on the SOI.
- Land cover (LC) map: represented as a categorical variable and developed by the SOI. Nine LC categories were defined: bare soil, orchards, water, crop fields, forest, natural grove, general vegetation, bare rock, and urban areas (Ohana‐Levi et al., 2018).

Linear autocorrelation was tested between variables before they were included in the Maxent models. The different models were run with up to 5,000 iterations and 15 replicates for each model. For each replicate, all presence data were divided into training panel (75%) and test panel (25%). The accuracy of the final model was estimated by computing the area under the curve (AUC) of the receiver operating characteristic (ROC) curve (Hanley & McNeil, 1982). The logistic output format of Maxent is a probability map that can be interpreted as the predicted probability for the occurrence of wild grapevines in the studied area. The resulting model was visualized as a map using the average value across 15 replications.

### 2.4 Phenotyping analysis

Among the sampled accessions, 68 plants from different sampling locations that were physiologically fit for phenotyping were morphologically characterized following the International Organization of Vine and Wine (OIV) standards adopted by the “COST Action GrapeNet FA1003” (2007). The list of 20 OIV descriptors that were used in this study is provided in Table S1.

A principal component analysis (PCA) was performed for the morphological characteristics using the “Gifi” package (de Leeuw & Mair, 2009) in R, which implements categorical PCA (“PRINCALS”) and is visualized using “ggplot2” package (Wickham, 2016). A comparison of the scores across all 20 OIV descriptors was conducted between the two populations, using the Wilcoxon signed‐rank test.

### 2.5 DNA extraction and genotyping

Total DNA was extracted using a modified cetyl‐trimethylammonium bromide (CTAB) protocol, described previously by Drori et al. (2017) and Drori, Rahimi, et al. (2017). Briefly, frozen leaves were weighed and ground with a pestle. CTAB buffer was added before incubation (65°C, 30 min) in a dry bath and stirred with vortex 3–4 times during incubation. An equal volume of chloroform:isoamyl alcohol (24:1) was added and mixed by inverting tubes. After phase separation, DNA was precipitated by 1/2 volumes of 5 M NaCl and 2 volumes of absolute cold ethanol. The extracted DNA pellets were air‐dried at room temperature and dissolved in 70 µl DNase‐free water (Promega Ltd., USA). Samples were stored in a −20°C freezer until genotyping.

Genotyping of each accession was conducted using a panel of 22 standard SSR markers that were developed for genotyping grapevine (Emanuelli et al., 2013). Two markers (VVMD5 and VVIn73) had high level of missing data and were excluded from the analyses. Multiplex polymerase chain reaction (PCR) amplifications were performed in a final volume of 25 μl containing 50 ng genomic DNA, 12.5 μM Go Taq Green Master Mix (Promega, USA), and 0.4 μM of each primer. The PCR products, including the GeneScan™ 500 ROXW size standard (Thermo Fisher Scientific Ltd., USA), were denatured and size‐fractionated using capillary electrophoresis on an ABI 3500 Genetic Analyzer (Thermo Fisher Scientific Ltd., USA). Finally, allele size estimation at each marker was determined using the software GeneMapper 5 (Thermo Fisher Scientific Ltd., USA).

### 2.6 Genetic diversity and population structure

To determine the number of populations and the assignment of samples to clusters, we used the Bayesian clustering analysis implemented in STRUCTURE 3.2 (Pritchard et al., 2000) with both the admixed and correlated allele frequency models. Twenty independent runs were conducted for each number of clusters (*K*) ranging from 1 to 9 using 50,000 iterations following a burn‐in length of 5,000 iterations. The most likely number of clusters was determined based on the log‐likelihood score of each *K* and the Δ*K* method (Evanno et al., 2005) using its implementation in the CLUMPAK software (Kopelman et al., 2015). For visualization, bar plots were generated with the Structure plot V2.0 interactive web application (Ramasamy et al., 2014).

Next, a neighbor‐joining tree was constructed using the genetic information obtained from all accessions in addition genetic data generated for 8 rootstock varieties (*Vitis rupestris*) which were included as an outgroup. The dendrogram was generated from a Bruvo's genetic distance (Bruvo et al., 2004) calculated with 1,000 bootstrap replicates using the R package poppr (Kamvar et al., 2014).

Analysis of genetic variation within and between the identified clusters using the analysis of molecular variance (AMOVA) and PCoA (principal coordinate analysis) was calculated as implemented in GenAlex v6.501 (Peakall & Smouse, 2012). The level of significance was computed based on 999 permutations. In addition, population genetics statistics were calculated for each cluster and included the fixation index (*F*), the observed heterozygosity (*H_o_*), unbiased expected heterozygosity (*H_e_*), the number of effective alleles (*N_e_*), Hardy–Weinberg equilibrium test (*HWE*), and the number of private alleles, extent of gene flow (*Nm*), and genetic differentiation (*F_ST_*) (Meirmans & Hedrick, 2011) using the GenAlex v6.501 software (Peakall & Smouse, 2012). Estimation of null allele frequencies was conducted with package “PopGenReport” (Adamack & Gruber, 2014) in R. A heatmap plot for the pairwise *F_ST_* matrix was generated using “ggplot2” package (Wickham, 2016) in R.

R scripts used in this study are provided as Supplementary R script file.

## 3 RESULTS

In a comprehensive survey conducted between 2017–2019, a wide range of habitats was screened from the Negev desert in the south to the Upper Galilee in the north of Israel to complement previous efforts to identify wild grapevine populations (Drori, Levy, et al., 2017; Drori et al., 2017; Drori, Rahimi, et al., 2017). This survey resulted in a total of 129 accessions which were identified and sampled for genetic analysis, albeit geographic locations were recorded only for 119 accessions (Figure 1b). All *V. v*. *sylvestris* populations identified in the survey originated from the Sea of Galilee catchment area, northern Israel. The population occurs in four distinct geographic subareas: (a) the northeastern Sea of Galilee shore (Betiha), a low‐altitude (approximately −200 m mean sea level (MSL)) flood plain, wherein several streams, including the Jordan River, flow into the lake and thick soils (mainly haploxerolls and vertisols) cover an extensive area, (b) the Upper Jordan River and its tributaries (circa 100 m MSL), where the grapevines are found mainly in basaltic haploxerolls, (c) the central part of the Golan Heights basaltic plateau (about 500 m MSL), and (d) the southern slops of the Golan Heights (around 300 m MSL), which are covered by haploxerolls, basaltic haploxerolls, and vertisols (Figure 1). In all those areas, precipitation similarly increases with elevation and natural streamflow toward the lake is calculated to be around 50% of total rain volume (Shentsis et al., 2018). Hence, even in lower altitudes where precipitation is lower, streamflow is suggested to significantly contribute to plant water availability. No *V. v*. *sylvestris* populations were found south of the Semech Creek near the Sea of Galilee (32°49′38.5″N 35°39′42.8″E); thus, these coordinates mark the global southern distribution edge of this species in accordance with previous survey conducted in this region (Drori et al., 2017; Drori, Rahimi, et al., 2017).

### 3.1 Multivariate spatial clustering

**FIGURE 2:**
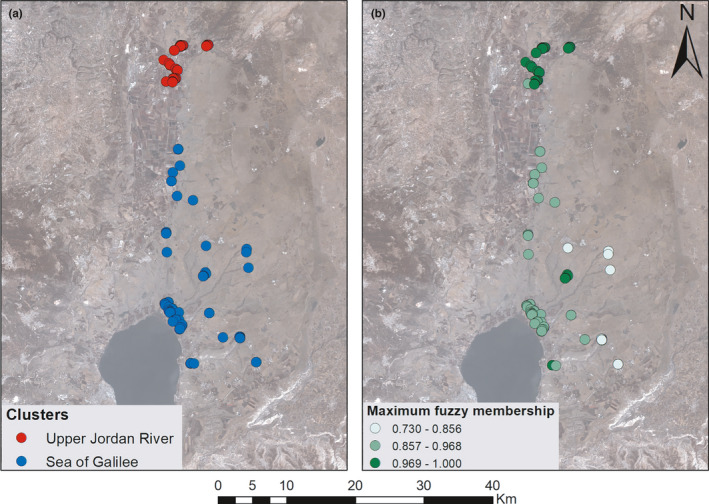
Multivariate spatial clustering according to seven environmental variables. (a) The grapevine locations assigned to the two clusters. (b) Maximum fuzzy membership values for each of the grapevines

Seven spatially environmental variables were included in the multivariate spatial clustering analysis conducted for the 119 accessions (Figure 2). The optimal number of clusters identified in the collection was obtained at two (silhouette index = 0.85), distinguishing between the two broadly defined ecogeographic regions, that is, the Upper Jordan River, and the Sea of Galilee which included also the Golan Heights (Figure 2a). The Upper Jordan River in the northern part of this region included 51 accessions and had higher membership values (membership > 0.968). The Sea of Galilee & Golan cluster consisted of 68 accessions and showed lower and more variable membership values (0.73 < membership < 0.989) implying that this region may represent a more complex ecogeographic structure (Figure 2b). As higher membership values indicate stronger similarity within the cluster, thus the Upper Jordan River region is less variable and better defined than the Sea of Galilee–Golan region.

### 3.2 Genetic differentiation between wild grapevine populations

The SSR markers used to test the genetic diversity in the collection were developed specifically to characterize grapevines and are evenly spread along 17 out of 19 linkage groups in the *Vitis vinifera* genome; thus, tight linkage disequilibrium is unlikely for this set of markers (Table S2). A null allele test was conducted for each marker and pointed that one locus (VVIv37) has a marginally high frequency of null alleles (0.22) compared to the acceptable frequency of 0.2 (Dakin & Avise, 2004). Next, the Hardy–Weinberg Equilibrium (HWE) was tested for each marker across all accessions and indicated that among the 20 SSRs in the study, 8 were at equilibrium, 3 were marginally accepted, and for the remaining markers, the null hypothesis of HWE was rejected (Table S2). These results indicate that at least half of the markers are affected by violations of the HWE assumptions and that the collection may be comprised of several distinguished populations with differences in allele frequencies. To test this hypothesis and identify the number of clusters that best represent the wild grapevine collection, a Bayesian clustering analysis was conducted in STRUCTURE using the SSR markers genotyped over all accessions. We used the Δ*K* test and the log‐likelihood scores for each *K* to determine the number of clusters and found that *K* = 2 (Δ*K* = 92.12) is the most probable number of clusters while at *K* = 3 (Δ*K* = 4.32) is the second‐best result (Figure S2a). Overall, accessions were assigned in accordance with their geographic sampling location, that is, the Upper Jordan River region and the Sea of Galilee region (Figure 1). At *K* = 3, the Sea of Galilee cluster was further split to a subpopulation at the south of Golan Heights which is highly isolated geographically and small (Figure S2b). Several individuals that were sampled at the intermediate geographic region between the Sea of Galilee and the Upper Jordan River had an admixed ancestry pattern; thus, gene flow is not prevented between the two identified clusters (Figure S2b).

A McNemar's χ^2^ test was applied to evaluate the similarity between the clustering pattern obtained by the genetic clustering and the multivariate spatial clustering. The results showed no significant difference (χ^2^ = 3.2, *p*‐value = .07) between the genetic and ecogeographic clustering indicating that the genetic differentiation closely corresponds to the environmental differences between regions.

**FIGURE 3:**
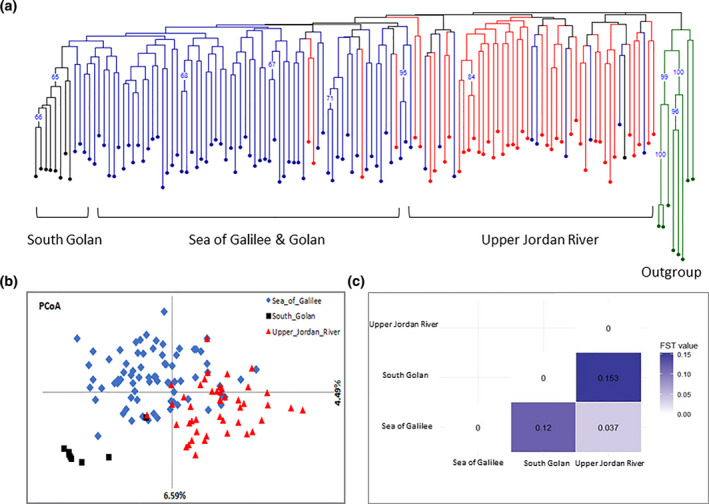
Population analysis of the *V. v*. *sylvestris* germplasm collection. (a) A neighbor‐joining dendrogram, based on Bruvo's genetic distance matrix calculated from the dataset of 20 SSR across 129 genotypes. Red dots—accessions collected in the Upper Jordan River; Blue dots—accessions collected at the Sea of Galilee; Black dots—accessions collected in the south‐Golan; Green dots—outgroup (rootstocks). (b) Principal coordinates analysis (PCoA) plot conducted via covariance matrix with data standardization for the 3 regional groups: Sea of Galilee (blue), south‐Golan (black), and Upper Jordan River (red). (c) A heatmap of pairwise FST values conducted between each pair of the 3 regional subpopulations

These results were further supported by a neighbor‐joining (NJ) dendrogram constructed across all accessions based on the 20 SSR markers (Figure 3a). The generally observed pattern in the NJ tree was to two major clusters (not supported by high bootstrap values) in accordance with the geographic location at the Upper Jordan River and the Sea of Galilee, except 13 accessions that were grouped not at their geographic group. However, some indications for a hierarchical clustering were observed in both major clades including a separated clade to the south‐Golan subpopulation inside the Sea of Galilee clade. A PCoA conducted using the same set of 20 SSR markers indicated similar pattern of clustering (Figure 3b). The first two principal components explained 11.08% of the variation in the analysis and distinguished between two main groups with some overlap. In the PCoA, the south‐Golan subpopulation was clearly separated from the Sea of Galilee cluster implying this subpopulation maintains a distinct genetic makeup from the rest of the cluster.

Next, an AMOVA was applied to test the genetic differentiation between the identified clusters at the Sea of Galilee and Golan and the second cluster at the Upper Jordan River area. The total genetic variation observed in the wild grapevine collection is attributed to differences between clusters (4%), compared to the variation within clusters (96%). We further calculated the level of genetic differentiation (*F_ST_*) between clusters which was found to be low (0.038), yet significant (*p*‐value = .001) indicating that the two clusters maintain some level of gene flow despite their clear split. To explore the structure and interactions between populations, genetic differentiation (*F_ST_*) and gene flow (*Nm*) were calculated for the two main clusters and also for the south‐Golan subpopulation (Table S3). The Upper Jordan River population and the Sea of Galilee clusters were slightly differentiated (*F_ST_* = 0.037) presumably due to extensive gene flow (*Nm* = 6.5666). The south‐Golan subpopulation was significantly differentiated (*F_ST_* = 0.153, 0.120), and low gene flow was obtained (*Nm* = 1.379, 1.832) with both the Upper Jordan River and the Sea of Galilee clusters (Figure 3c). These results were further supported by the number of private alleles indicating that the south‐Golan subpopulation had 9 private alleles compared with 17 and 14 private alleles in the Upper Jordan River and the Sea of Galilee, respectively, although the last two clusters include 9.5 and 5.5 times the number of accessions, respectively (Table S4).

To study the genetic diversity among the identified clusters, the level of observed (*H_o_*) and expected (*H_e_*) heterozygosity and inbreeding coefficients (*F*) were calculated. A high level of genetic diversity (*H_o_* = 0.766, *F* = −0.006) was observed in the Upper Jordan River cluster compared with the Sea of Galilee cluster which was characterized by higher inbreeding coefficient *(F* = 0.091) and lower observed heterozygosity (*H_o_* = 0.677). Conducting the same analysis while separating into the three subpopulations mentioned above, the south‐Golan subpopulation showed the lowest heterozygosity (*H_o_* = 0.613) and fixation index (*F* = −0.16). (Table 1).

**TABLE 1:** Summary of genetic variation statistics at 20 SSR loci in the *V. v*. *sylvestris* germplasm collection for two structure divisions (*K* = 2, *K* = 3)

*K* = 2		*N*	*Na*	*Ne*	*Ho*	*He*	*F*
Sea of Galilee	Mean	84	10.750	5.048	0.677	0.748	0.091
	*SE*		0.917	0.501	0.032	0.032	0.022
Upper Jordan River	Mean	45	8.800	4.583	0.766	0.763	−0.006
	*SE*		0.592	0.293	0.020	0.017	0.019

*K* = 3		*N*	*Na*	*Ne*	*Ho*	*He*	*F*
Sea of Galilee	Mean	76	11.050	5.203	0.661	0.758	0.122
	*SE*		0.905	0.503	0.029	0.030	0.026
South‐Golan	Mean	8	4.150	2.627	0.613	0.528	−0.160
	*SE*		0.437	0.296	0.060	0.049	0.063
Upper Jordan River	Mean	45	9.300	4.779	0.746	0.771	0.029
	*SE*		0.633	0.317	0.023	0.018	0.027

Abbreviations: *F*, fixation index (inbreeding coefficient); *He*, unbiased expected heterozygosity; *Ho*, observed heterozygosity; *N*, sample size; *Na*, mean number of alleles per locus; *Ne*, number of effective alleles; *SE*, standard error.

### 3.3 Morphologic differentiation between wild grapevine populations

**FIGURE 4:**
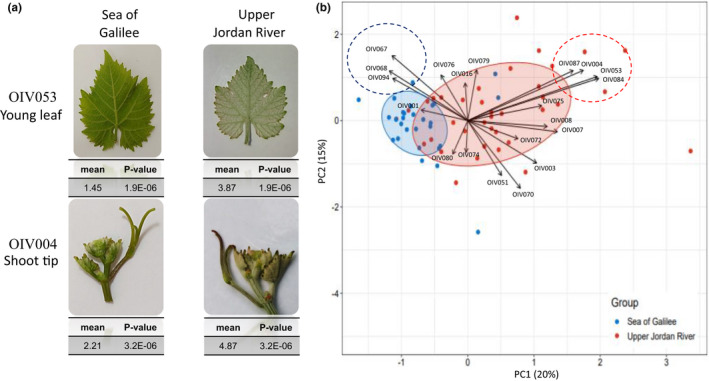
Phenotypic analysis of the *V. v*. *sylvestris* population. (a) Photographs representing the differences in the density of prostrate hairs on young leaves (OIV053—density of prostrate hairs between main veins on the lower side of the blade) and young shoots (OIV004—density of prostrate hairs on the shoot tip) of wild grapevines of the Sea of Galilee and Upper Jordan River subpopulations. The means and *p*‐value presented were calculated by the Wilcoxon test comparing the Sea of Galilee and Upper Jordan River subpopulations. (b) PCA biplot of an analysis performed on the 68 *V. v*. *sylvestris* accessions, using 20 OIV descriptors (colored ellipses represent 70% probability)

Phenotypic analysis was conducted for 68 individuals representing the Upper Jordan River (*n* = 39) and Sea of Galilee (*n* = 29) regions using 20 OIV descriptors (Figure 4a; Table S1). A PCA was conducted using all 20 OIV descriptors (Figure 4b), and the first two principal components explained 35% of the variation in the analysis and were mostly affected by the prostrate hairiness (OIV004, OIV053, and OIV084), erect hairiness (OIV087), shape of blade (OIV067), number of lobes (OIV068), and depth of upper lateral sinuses (OIV094). Overall, the individuals from the Upper Jordan River are characterized by higher leaf hairiness while the individuals from the Sea of Galilee are characterized by leaves with the higher number of lobes (Figure 4b). To validate the interpretation of the PCA, a Wilcoxon signed‐rank test was conducted for the hairiness descriptors (OIV004, OIV053, and OIV084) and the number of lobes scores (descriptor OIV068). The results of the test supported the PCA indicating that the Upper Jordan River cluster is characterized by significantly higher hairiness (*p*‐value < .005) and lower number of lobes (*p*‐value = .003) than that of the Sea of Galilee subpopulation (Table S5).

### 3.4 Environmental factors affecting wild grapevine distribution

**FIGURE 5:**
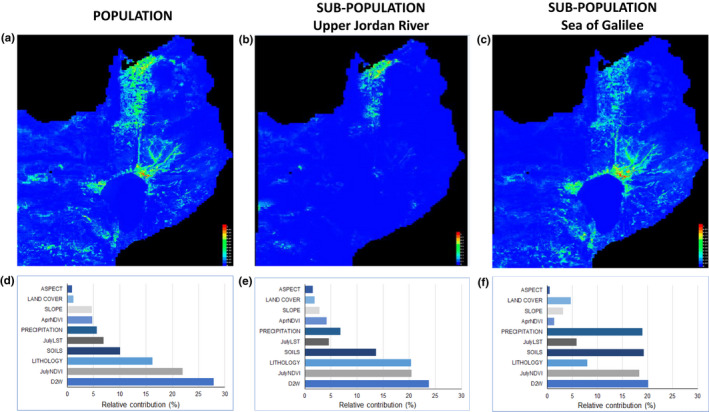
Prediction of the *V. v*. *sylvestris* distribution in the South Levant and its affecting factors. Panels (a‐c) are probability maps of predicted habitat suitability for *V. v*. *sylvestris*, determined using the (a) whole population (Upper Jordan River and Sea of Galilee subpopulations), (b) Upper Jordan River subpopulation separately, and (c) the Sea of Galilee subpopulation separately. Panels (d‐f) show the relative contributions of environmental variables obtained from a Maxent analysis for the (d) entire population, (e) Upper Jordan River, and (f) Sea of Galilee: “ASPECT” is the slope aspect (°); “LAND COVER” represents different land cover categories; “SLOPE” is the hillslope (°); “AprNDVI” is NDVI calculated using a satellite image acquired in April 2017; “PRECIPITATION” is the mean annual precipitation (mm); “JulyLST” is the land surface temperature calculated using a satellite image acquired in July 2017 (°C); “SOILS” is a variety of soils categories; “LITHOLOGY” is a variety of lithology categories; “JulyNDVI” is the NDVI calculated using a satellite image acquired in July 2017, and “D2W” is the distance from water bodies (in meters)

To explore the contribution of environmental factors to the occurrence of wild grapevine at the south edge of its distribution range, a niche modeling analysis was conducted. The analysis was performed to predict the habitat suitability using the information at the grid cell resolution for each of the two main subregions identified in the multivariate spatial clustering: Upper Jordan River, Sea of Galilee, and for the entire study area (Figure 5). Overall, the obtained models had high prediction accuracy ranging between AUC values of 0.93–0.97.

Among the ten environmental variables used to calculate the three models, distance to water was the strongest contributor (entire region—27.8%, Upper Jordan River—23.7%, and Sea of Galilee—20.1%) to wild grapevine spatial distribution across the studied area (Figure 5d‐f, Table S6). Next in rank was the NDVI in July which explained 21.9%, 20.4%, and 18.3% of the model for the entire area, the Upper Jordan River region, and the Sea of Galilee region, respectively. In the entire studied area and Upper Jordan River region lithology was the third most contributing factor explaining 16.2%, and 20.3% of the variation in the model, respectively. In the Sea of Galilee region, soil type (19.2%) and precipitation (18.9%) were the next contributing variables to the model.

**FIGURE 6:**
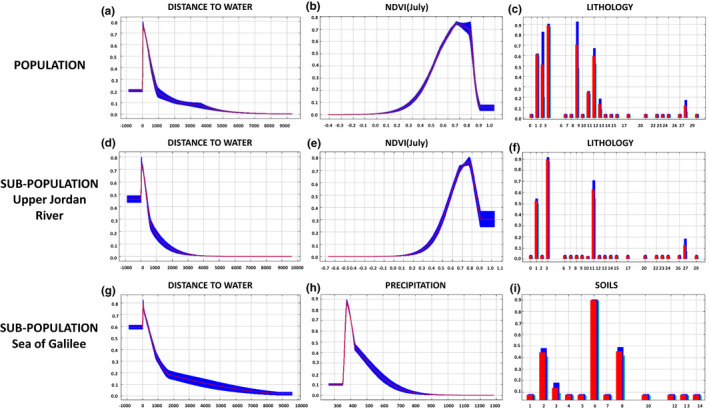
Response curves of the three most contributing variables for the entire region (all), Upper Jordan River, and Sea of Galilee subpopulations. The bars and curves show the mean response of the 15 replicates of Maxent runs (red) and the mean ± standard deviation (blue). These plots reflect the dependence of the predicted probability of *V. v*. *sylvestris* habitation on the selected variable

In accordance with the contribution of the environmental factors to wild grapevine distribution, the predicted habitat suitability was found to decrease in probability at distances higher than 1 km from water sources and found higher at July NDVI levels between 0.6 and 0.8, and precipitation range of 375–410 mm per year (Figure 6). Habitats with mean annual precipitation lower than 350 mm per year were found to be unsuitable for the occurrence of stable *V. v*. *sylvestris* populations throughout the studied area. Outcropping lithology was found to affect the occurrence of wild grapevine only at the Upper Jordan River region, while the soil type was found to affect the occurrence only at the Sea of Galilee region, and the high occurrence probability was obtained in areas characterized by vertisols (rich clay soils) in both regions (Tables S7 and S8). All other tested variables had a low contribution to the distribution of wild grapevine in the studied area.

## 4 DISCUSSION

What limits the distribution of species is a fundamental question in ecological and evolutionary biology. Wild grapevine is a perennial species of economic importance due to its value for breeding purposes in cultivated grapevines which are among the most important horticulture crops in the world. We established a new wild grapevine germplasm collection comprised of 129 accessions that represent the south‐most natural populations along the distribution range of this species. This collection was characterized genetically and phenotypically, and the obtained information was compared to available environmental information for each sampling location.

### 4.1 Wild grapevine population structure is determined by genetic and ecogeographic factors

Wild grapevine populations were identified at four distinct geographic regions in northern Israel. These regions are characterized by different environmental conditions including altitude, temperature, and precipitation. The analyses of the ecogeographical data suggested that the collection can be generally divided into two mains regions: The Upper Jordan River and the Sea of Galilee which include also the Golan Heights. This split was further supported by most of the genetic analyses, including the neighbor‐joining tree and the STRUCTURE analysis indicating that the differentiation to clusters is the outcome of both ecological and evolutionary processes. Expectedly, the split to distinct genetic clusters coincide with the ecogeographical discrimination to regions as indicated by McNemar's χ^2^ test. Interestingly, the maximum fuzzy membership analysis indicated higher membership assignment of individuals to the Upper Jordan River region while the individuals assigned to the Sea of Galilee region are more variable in the level of assignment (Figure 2b). These results suggest that unlike the clear boundaries definition for the Upper Jordan River region, the Sea of Galilee region may actually represent a mixture of ecogeographical regions.

Indeed, the Sea of Galilee region as defined in the multivariate spatial clustering and the STRUCTURE analyses include the individuals collected at the Golan Heights which is characterized by higher altitude and distinguished soil and climate conditions compared with the region around the Sea of Galilee. Despite the fact that the split to two clusters were supported by both ecogeographic and genetic analyses, some indications imply that that the Golan Heights subpopulation should be treated separately from the Sea of Galilee cluster. These indications were also supported by the population genetics analyses as detailed below. The sensitivity of STRUCTURE to distinguish between populations relies on its ability to identify differences in the allele frequency spectrum of each population (Porras‐Hurtado et al., 2013). Small sample size, as in the case of the south‐Golan subpopulation, may bias the frequency spectrum and diminish the ability to differentiate it from the large Sea of Galilee cluster. In addition, Cullingham et al. (2020) recently described that a strong bias toward *K* = 2 occurs when using the Δ*K* method.

The pairwise population comparisons of *F_ST_* and *Nm* values could be seen as an indirect estimate of isolation and migration between populations (Slatkin, 1987). The results of the *F_ST_* and *Nm* statistics between the Upper Jordan River and Sea of Galilee clusters indicated that these two populations are differentiated, even though some level of gene flow remains (Table S3). Moreover, the higher genetic diversity in the Upper Jordan River, as estimated by the number of alleles and expected heterozygosity, may imply that this population is ancestral to the Sea of Galilee population. It is tempting to speculate that the different ecogeographic conditions in each region maintain these two populations differentiated, as indicated by both phenotypic and genetic data, through selection. Sadly, the available genetic data in the study did not allow to test this hypothesis explicitly with high confidence. However, the observed heterozygosity was relatively high across regions (0.61–0.75, Table 1), similar to those reported for the Mediterranean basin and Central Asia wild populations (Riaz et al., 2018), indicating that overall, high genetic diversity is preserved among the wild populations in the south Levant.

Testing the level of differentiation and gene flow between the south‐Golan and each of the main clusters indicated that this subpopulation is distinguished. The observed high *F_ST_*, low *Nm*, low heterozygosity yet relatively high number of private alleles in this subpopulation indicate this is a newly established or highly isolated subpopulation which has emerged from the Sea of Galilee cluster or from a different source (Table S3). The PCoA conducted based on the available genetic data further supported the separation of the south‐Golan subpopulation from the Sea of Galilee and Upper Jordan River subpopulation clusters.

Considering all the above results, we propose the following possible species dynamics and distribution model: The initial population was established at the Upper Jordan River region, which borders other wild grapevine populations at the north (Lebanon) and east (Syria). The Sea of Galilee population, which marks the southernmost local and global edge of wild grapevine distribution range (Rottenberg, 1998), likely originated from the Upper Jordan River, with continuous gene flow preserved between the two regions. The south‐Golan subpopulation is presumably a distinct population which has emerged recently from the Sea of Galilee population. The south‐Golan subpopulation is an isolated small group; thus, low gene flow with the other population was observed although it could not be separated from the Sea of Galilee population based on the ecogeographical and STRUCTURE analyses. Another potential explanation could be that the south‐Golan subpopulation has originated from Syria on the east. The location of this population, its isolation, and its negative fixation index (indicating excess outbreeding) seem to support this hypothesis although the results obtained from the analyses conducted do not conclusively support this.

### 4.2 Phenotypic differentiations between the different subpopulations

The analysis of the measured morphology across individuals well supported both the genetic and ecogeographic results of differentiation between the two clusters at the Upper Jordan River and the Sea of Galilee. The main phenotypic characteristics that were differentiated between the two populations are the hairiness traits on both shoot tip and leaves (Figure 4b). Hairiness has an important contribution to both biotic and abiotic stress resistance in plants. For example, a study on grapevine response to *erineum* mite found that leaf hairiness, leaf wax, and carbohydrate content are strongly associated with resistance (Khederi et al., 2014). Another study conducted in *Sinapis arvensis* suggested that the level of hairiness can have a contribution to plant fitness under both biotic and abiotic stress and in different environments (Roy et al., 1999). The differences observed between the Upper Jordan River and the Sea of Galilee in the level of hairiness imply that the differentiation to distinguished clusters is indeed associated with the different environmental conditions which pose a selective constrain for population admixture. However, it is unclear whether the low‐hairiness phenotype observed in the Sea of Galilee population is heritable or epigenetic. Further analysis of the phenotypes under controlled conditions is necessary to address this open question.

### 4.3 The ecogeographic factors constraining wild grapevine distribution

A central aspect in the study of the ecology and evolution of wild grapevine is what are the ecogeographic constraints for its distribution, specifically at the Levant which marks this species southern boundaries. The main environmental factors that affect the occurrence of wild grapevine in the studied area were availability of flowing water as indicated in the MAXENT model. This observation was supported by both the distance to water source and NDVI parameters throughout the studied area and for each region separately (Figure 5).

The NDVI in July represents the biomass and density of vegetation (Cabrera‐Bosquet et al., 2011), which can only be maintained throughout the dry summer in the southern Levant in areas surrounding water sources or irrigated plots. Indeed, NDVI was found to be an indicator of water availability for woody vegetation (Aguilar et al., 2012). In contrast, in areas with denser vegetation during the summer, wild grapevine occurrences decreased dramatically. In the south Levant area, the highest vegetation index levels during the summer were observed in cultivated orchards and fields, indicating that intensive agricultural manipulation hinders wild grapevine distribution. Open areas in this region are being increasingly transformed into farmed lands and urban regions. Some riverbanks are routinely sprayed with herbicides or mowed, bringing the wild grapevine in this region to the brink of extinction. Thus, our findings urge an active conservation of wild grapevines in the southern Levant.

At the Sea of Galilee region, precipitation was found to be the next most important limiting factor for the distribution of wild grapevine. The Sea of Galilee region is at the border of the semi‐arid region at the southern Jordan Valley where precipitation drastically decreases southwards. Thus, in years of low precipitation or drought, the Sea of Galilee region suffers from low water availability compared with the Upper Jordan River region where springs and water streams coming from northern regions are flowing also in drought years.

Considering these results and observations, we conclude that wild grapevine populations in the southern Levant area are not adapted to drought conditions and can occur mainly along flowing water sources or active springs. In the few cases where wild grapevine plants were found distant from the water source, they were growing on fences of regularly irrigated fruit tree orchards. This is in accordance with a previous study which showed that wild grapevine populations distribution in Italy was highly associated with the hydrographic network (Biagini et al., 2014). Similar observations were also reported in Spain, Georgia, and Portugal, where wild grapevines were mostly found along river banks (Cunha et al., 2007; Ekhvaia & Akhalkatsi, 2010; Ocete, Muñoz‐Organero, et al., 2011).

The outcropping lithology and the soil composition had an impact of secondary importance on the occurrence of wild grapevine across the studied area (Figure 6f,i). The results of our analyses indicated that wild grapevine usually avoids the outcropping basalts and prefers regions where haploxerolls are developed in carbonate rocks and where vertisols cover colluvial beds. These findings support previous studies, which found that the colluvial substratum and alluvial soils are preferred by wild grapevine populations in Italy (Biagini et al., 2014).

An interesting prediction of the Maxent model was that wild grapevine populations should be present in a small area at the western shore of the Sea of Galilee (Figure 5a). A thorough survey conducted in the area did not yield any findings of an existing or remains of established population at this location. One explanation for this erroneous prediction of the model is the lack of a detailed soil salinity GIS layer for this region. High soil salinity at the western coast of the Sea of Galilee is a well‐documented phenomena and occurs due to saline springs located in the area (Gvirtzman et al., 1997; Rimmer & Nishri, 2014). Hence, salinity may be an important factor affecting the soil composition and restrict the wild grapevine distribution in this region.

## 5 CONCLUSIONS

To better understand the ecology and evolution of the wild grapevine, we studied the demographic and environmental factors limiting the occurrence of populations at the south edge of the species distribution range. The results of the environmental, genetic, and phenotypic analyses supported the division to two main clusters: one at the Upper Jordan River and another around the Sea of Galilee. Interestingly, the two main clusters were also distinguished are the ecogeographic conditions, in addition to the hairiness morphology which imply that natural selection maintains the differentiation to populations despite extensive gene flow between them. Nevertheless, several lines of evidence support a split in the Sea of Galilee population to a distinguished isolated subpopulation at the south‐Golan region. We suggest that the Upper Jordan River population is ancestral to the Sea of Galilee population and that the south‐Golan subpopulation may have split recently from the Sea of Galilee population or from another unsampled population in this region. The main ecogeographic factors constraining the distribution of wild grapevine at its southern distribution range are proximity to water sources, NDVI levels, and sufficient precipitation rates, specifically at the southern populations that are closer to the semi‐arid condition in the south. To the best of our knowledge, the Sea of Galilee population marks the southernmost occurrence of wild grapevine which is limited by water availability. Our results emphasize the importance of recording, sampling, and conserving wild grapevine populations which are threatened by intensive anthropogenic activity in this region, in addition to climatic turnovers.

## CONFLICT OF INTEREST

The authors declare that they have no conflict interests.

## AUTHOR CONTRIBUTIONS

**Oshrit Rahimi:** Investigation (equal); project administration (supporting); resources (supporting); software (lead); validation (lead); visualization (lead); writing‐original draft (equal). **Noa Ohana‐Levi:** Conceptualization (supporting); data curation (supporting); formal analysis (supporting); investigation (supporting); resources (supporting); software (equal); validation (supporting); visualization (equal); writing‐original draft (supporting). **Hodaya Brauner:** Conceptualization (supporting); formal analysis (supporting); resources (equal); Visualization (supporting). **Nimrod Inbar:** Data curation (supporting); software (supporting); writing‐original draft (supporting). **Sariel Hübner:** Conceptualization (equal); investigation (supporting); methodology (supporting); resources (supporting); software (supporting); supervision (equal); writing‐original draft (equal). **Elyashiv Drori:** Conceptualization (lead); data curation (supporting); formal analysis (supporting); funding acquisition (lead); investigation (equal); methodology (equal); project administration (supporting); resources (supporting); supervision (lead); validation (supporting); visualization (supporting); writing‐original draft (equal).

## Supporting information

Supplementary Material

Supplementary Material

## Data Availability

The Maxent input files, morphological and microsatellite data sets are available at the associated Dryad repository: https://doi.org/10.5061/dryad.k3j9kd56p (Rahimi et al., 2021).
