# Supplementary material for: Demographic and ecogeographic factors limit wild grapevine spread at the southern edge of its distribution range
Source: Ecol Evol. 2021 May 8;11(11):6657–71. doi: 10.1002/ece3.7519 (PMC8207413; doi:10.1002/ece3.7519)
Supplement: Supplementary file 1 — Supplementary Material [file ECE3-11-6657-s001.docx]

**Supplementary Information**

**Demographic and ecogeographic factors limit wild grapevine spread at the southern edge of its distribution range**

Rahimi Oshrit^1^, Ohana-Levi Noa^2^, Brauner Hodaya^3^, Inbar Nimrod ^4,5^, *Hübner Sariel^6^ and *Drori Elyashiv^1, 3^

**Table S1.** Summary of the 20 OIV ampelographic descriptors used in this work, in accordance with the OIV standards adopted by the "COST Action GrapeNet FA1003" (2007).

| **OIVCode** | **Ampelographic characteristics** |
| --- | --- |
| OIV001 | Opening of the shoot tip |
| OIV003 | Intensity of anthocyanin coloration on prostrate hairs of the shoot tip |
| OIV004 | Density of prostrate hairs on the shoot tip |
| OIV007 | Color of the dorsal side of internodes |
| OIV008 | Color of the ventral side of internodes |
| OIV016 | Number of consecutive tendrils on the shoot tip |
| OIV051 | Color of upper side of blade (young leaf) |
| OIV053 | Density of prostrate hairs between main veins on lower side of blade (young leaf) |
| OIV067 | Shape of blade (mature leaf) |
| OIV068 | Number of lobes (mature leaf) |
| OIV070 | Area of anthocyanin coloration of main veins on upper side of blade (mature leaf) |
| OIV072 | Goffering of blade (mature leaf) |
| OIV074 | Profile of blade in cross section (mature leaf) |
| OIV075 | Blistering of upper side of blade (mature leaf) |
| OIV076 | Shape of teeth (mature leaf) |
| OIV079 | Degree of opening / overlapping of petiole sinus (mature leaf) |
| OIV080 | Shape of base of petiole sinus (mature leaf) |
| OIV084 | Density of prostrate hairs between main veins on lower side of blade (mature leaf) |
| OIV087 | Density of erect hairs on main veins on lower side of blade (mature leaf) |
| OIV094 | Depth of upper lateral sinuses (mature leaf) |

**Table S2:** Summary of Hardy-Weinberg equilibrium test, Null alleles frequencies and Linkage groups for the SSR markers**.**

| **Locus** | **HWE** | | **Null alleles**  **(frequency)** | | **Linkage Group^a^** | |
| --- | --- | --- | --- | --- | --- | --- |
| VVMD27 | ns | 2.08E-4 | | 5 | |  |
| VVMD28 | *** | 0.090 | | 3 | |  |
| VVS2 | ns | 0.040 | | 11 | |  |
| VVMD7 | ns | 0.066 | | 7 | |  |
| VVMD32 | ** | 0.069 | | 4 | |  |
| VMC1b11 | * | 0.022 | | 8 | |  |
| VrZAG62 | ** | -0.013 | | 7 | |  |
| VVMD25 | ns | -0.026 | | 11 | |  |
| VrZAG79 | ns | 0.064 | | 5 | |  |
| VVMD24 | * | 0.046 | | 14 | |  |
| VVIn16 | ns | 0.004 | | 18 | |  |
| VVIq52 | ns | -0.020 | | 9 | |  |
| VVIh54 | *** | 0.052 | | 13 | |  |
| VVIv37 | *** | 0.220 | | 10 | |  |
| VMC4f8 | * | 0.049 | | 1 | |  |
| VVMD21 | *** | 0.049 | | 6 | |  |
| VMC4f3.1 | *** | 0.012 | | 12 | |  |
| VVIb01 | ns | 0.046 | | 2 | |  |
| VVIp31 | *** | 0.020 | | 19 | |  |
| VVIv67 | *** | 0.075 | | 15 | |  |

ns = not significant, * P<0.05, ** P<0.01, *** P<0.001 ^a^ Linkage groups are numbered according to Adam-Blondon et al. (2004), and Zarouri et al. (2015).

|  |
| --- |

**Table S3**. The calculation of the pairwise *F_ST_* values between populations (red) and pairwise *Nm* values between populations (black) for the south Levant *V.v. sylvestris* germplasm collection

|  | **Sea of Galilee** | **South Golan** | **Upper Jordan River** |
| --- | --- | --- | --- |
| **Sea of Galilee** | **-** | 0.120 | 0.037 |
| **South Golan** | 1.832 | **-** | 0.153 |
| **Upper Jordan River** | 6.566 | 1.379 | **-** |

**Table S4:** Summary of private alleles in three subpopulations

| **Sub-Population** | **Locus** | **Allele** | ***Frequency** |
| --- | --- | --- | --- |
| **Sea of Galilee** | VVMD28 | 247 | 0.021 |
|  | VVMD28 | 269 | 0.021 |
|  | VVS2 | 126 | 0.100 |
|  | VVS2 | 138 | 0.040 |
|  | VVS2 | 154 | 0.020 |
|  | VVMD7 | 235 | 0.034 |
|  | VVMD32 | 241 | 0.033 |
|  | VVMD32 | 247 | 0.013 |
|  | VMC1B11 | 181 | 0.110 |
|  | VVIQ52 | 86 | 0.013 |
|  | VVIH54 | 140 | 0.114 |
|  | VMC4F8 | 103 | 0.053 |
|  | VVIP31 | 177 | 0.142 |
|  | VVIV67 | 346 | 0.034 |
| **South Golan** | VMC1B11 | 163 | 0.063 |
|  | VrZAG79 | 241 | 0.125 |
|  | VVIH54 | 134 | 0.071 |
|  | VVIH54 | 170 | 0.071 |
|  | VVIV37 | 172 | 0.063 |
|  | VMC4F3.1 | 159 | 0.063 |
|  | VMC4F3.1 | 161 | 0.063 |
|  | VVIB01 | 297 | 0.063 |
|  | VVIV67 | 378 | 0.063 |
| **Upper Jordan River** | VVMD27 | 175 | 0.047 |
|  | VVS2 | 146 | 0.011 |
|  | VVMD7 | 259 | 0.022 |
|  | VVMD32 | 275 | 0.012 |
|  | VMC1B11 | 195 | 0.011 |
|  | VVMD25 | 244 | 0.044 |
|  | VVMD25 | 268 | 0.022 |
|  | VVIV37 | 164 | 0.013 |
|  | VVIV37 | 180 | 0.013 |
|  | VVMD21 | 251 | 0.045 |
|  | VVMD21 | 257 | 0.023 |
|  | VMC4F3.1 | 175 | 0.033 |
|  | VMC4F3.1 | 209 | 0.022 |
|  | VMC4F3.1 | 223 | 0.044 |
|  | VVIB01 | 299 | 0.033 |
|  | VVIP31 | 195 | 0.022 |
|  | VVIV67 | 370 | 0.026 |
|  |  |  |  |

*only alleles with Frequency > 0.01 are presented**.**

**Table S5.** Means obtained from 20 OIV descriptors and *p-value*s of Wilcoxon signed-rank test between Sea of Galilee and Upper Jordan River subpopulations

| **OIV** | **Sea of Galilee** | **Upper Jordan River** | **Wilcoxon signed-rank test** |
| --- | --- | --- | --- |
| **descriptor** | Mean | Mean | *p-value* |
| **OIV001** | 4.34 | 4.21 | 0.508 |
| **OIV003** | 1.86 | 2.62 | 0.191 |
| **OIV004** | 2.21 | 4.87 | **3.2E-06** |
| **OIV007** | 1.14 | 1.87 | **0.013** |
| **OIV008** | 0.93 | 1.05 | 0.458 |
| **OIV016** | 1.17 | 1.21 | 0.869 |
| **OIV051** | 1.59 | 2.03 | 0.130 |
| **OIV053** | 1.45 | 3.87 | **1.89E-06** |
| **OIV067** | 2.97 | 2.56 | 0.089 |
| **OIV068** | 2.59 | 1.95 | **0.003** |
| **OIV070** | 1.90 | 2.23 | 0.202 |
| **OIV072** | 1.38 | 1.87 | 0.255 |
| **OIV074** | 1.31 | 1.41 | 0.34 |
| **OIV075** | 1.69 | 3.38 | **2.63E-04** |
| **OIV076** | 4.21 | 4.05 | 0.601 |
| **OIV079** | 2.86 | 2.85 | 0.969 |
| **OIV080** | 2.03 | 1.87 | 0.462 |
| **OIV084** | 1.76 | 3.36 | **0.005** |
| **OIV087** | 1.69 | 2.31 | 0.164 |
| **OIV094** | 3.28 | 2.59 | 0.065 |

**Table S6.** Relative contributions of environmental variables as determined in a Maxent analysis

| **Environmental variable (unit)** | **Variable code** | **Entire population (%)** | **Upper Jordan River (%)** | **Sea of Galilee (%)** |
| --- | --- | --- | --- | --- |
| Distance to water (m) | D2W | 27.85 | 23.79 | 20.10 |
| NDVI at the month of July | JulyNDVI | 21.98 | 20.45 | 18.34 |
| Lithology categories | LITH | 16.21 | 20.34 | 7.97 |
| Soils categories | SOILS | 10.02 | 13.66 | 19.19 |
| LST at the month of July (°C) | JulyLST | 6.90 | 4.57 | 5.83 |
| Mean annual precipitation (mm) | PREC | 5.57 | 6.82 | 18.91 |
| NDVI at the month of April | AprNDVI | 4.74 | 4.19 | 1.35 |
| Slope (°) | SLOPE | 4.67 | 2.80 | 3.13 |
| Land cover categories | Land Cover | 1.13 | 1.88 | 4.70 |
| Aspect (°) | Aspect | 0.93 | 1.50 | 0.47 |

**Table S7.** Lithology types included in the lithology variable analysis of the probability of *V.v.* *sylvestris* occurrence (*P*), determined by a Maxent analysis.

| **Sea of Galilee**  ***(P)*** | **Upper Jordan River**  ***(P)*** | **Entire population (*P*)** | **Lithological categories** | **ID** |
| --- | --- | --- | --- | --- |
| 0.08 | 0.03 | 0.07 | Basalt and volcanic | 0 |
| **0.62** | **0.52** | **0.61** | Clay silt sand gravel | 1 |
| **0.67** | 0.03 | **0.49** | Landslide | 2 |
| 0.08 | **0.90** | **0.88** | Travertine | 3 |
| 0.08 | 0.03 | 0.07 | Sandstone | 6 |
| 0.08 | 0.03 | 0.07 | Marl very soft chalk | 7 |
| **0.58** | 0.03 | **0.71** | Sand gravel clay | 8 |
| 0.08 | 0.03 | 0.07 | Tuff scoria | 9 |
| **0.41** | 0.02 | 0.27 | Basalt | 10 |
| 0.08 | **0.63** | **0.59** | Conglomerate sandy | 11 |
| **0.27** | 0.03 | 0.12 | Sandstone gravel conglomerate limestone marl clay gypsum | 12 |
| 0.08 | 0.03 | 0.07 | Limestone and dolomite (inc. chert) | 13 |
| 0.08 | 0.03 | 0.07 | Sandstone clay conglomerate | 14 |
| 0.08 | 0.03 | 0.07 | Chalk | 15 |
| 0.08 | 0.03 | 0.07 | Limestone chalk and marl | 17 |
| 0.08 | 0.03 | 0.07 | Sea | 20 |
| 0.08 | 0.03 | 0.07 | Igneous and metamorphic | 22 |
| 0.08 | 0.03 | 0.07 | Chalk and marl | 23 |
| 0.08 | 0.03 | 0.07 | Chalk and chert | 24 |
| 0.08 | 0.03 | 0.07 | Chalk and limestone (inc. chert) | 26 |
| 0.08 | **0.13** | **0.12** | Limestone (inc. chert) | 27 |
| 0.08 | 0.03 | 0.07 | Limestone | 29 |

**Table S8.** Soil types included in the soil variable analysis of the probability of *V.v. sylvestris* occurrence (*P*), determined using a Maxent analysis.

| **ID** | **Soil categories** | **Entire population *(P)*** | **Upper Jordan**  **River**  ***(P)*** | **Sea of Galilee**  ***(P)*** |
| --- | --- | --- | --- | --- |
| 1 | Xero chrept, Haploxerolls, Xeror thents | 0.04 | 0.03 | 0.08 |
| 2 | Chromic Vertisols | **0.57** | **0.57** | **0.44** |
| 3 | Haplo xeralfs, Haploxerolls | 0.07 | 0.03 | 0.14 |
| 4 | Lithic Haploxerolls | **0.44** | **0.53** | 0.08 |
| 5 | Torrior thents, Haploxerolls | 0.04 | 0.03 | 0.08 |
| 6 | Dark Haploxeroll | **0.83** | 0.03 | **0.90** |
| 7 | Orthic Luvisols  (Brown Hamra) | 0.04 | 0.03 | 0.08 |
| 8 | Xero chrept, Haploxerolls, Chromo xerepts | **0.37** | 0.04 | **0.45** |
| 10 | Calcic Xerosols | **0.40** | **0.46** | 0.08 |
| 12 | Mixed | 0.04 | 0.03 | 0.08 |
| 13 | Lake | 0.04 | 0.03 | 0.08 |
| 14 | Regosols, Gypsi orthids, Camb orthids | 0.04 | 0.03 | 0.08 |


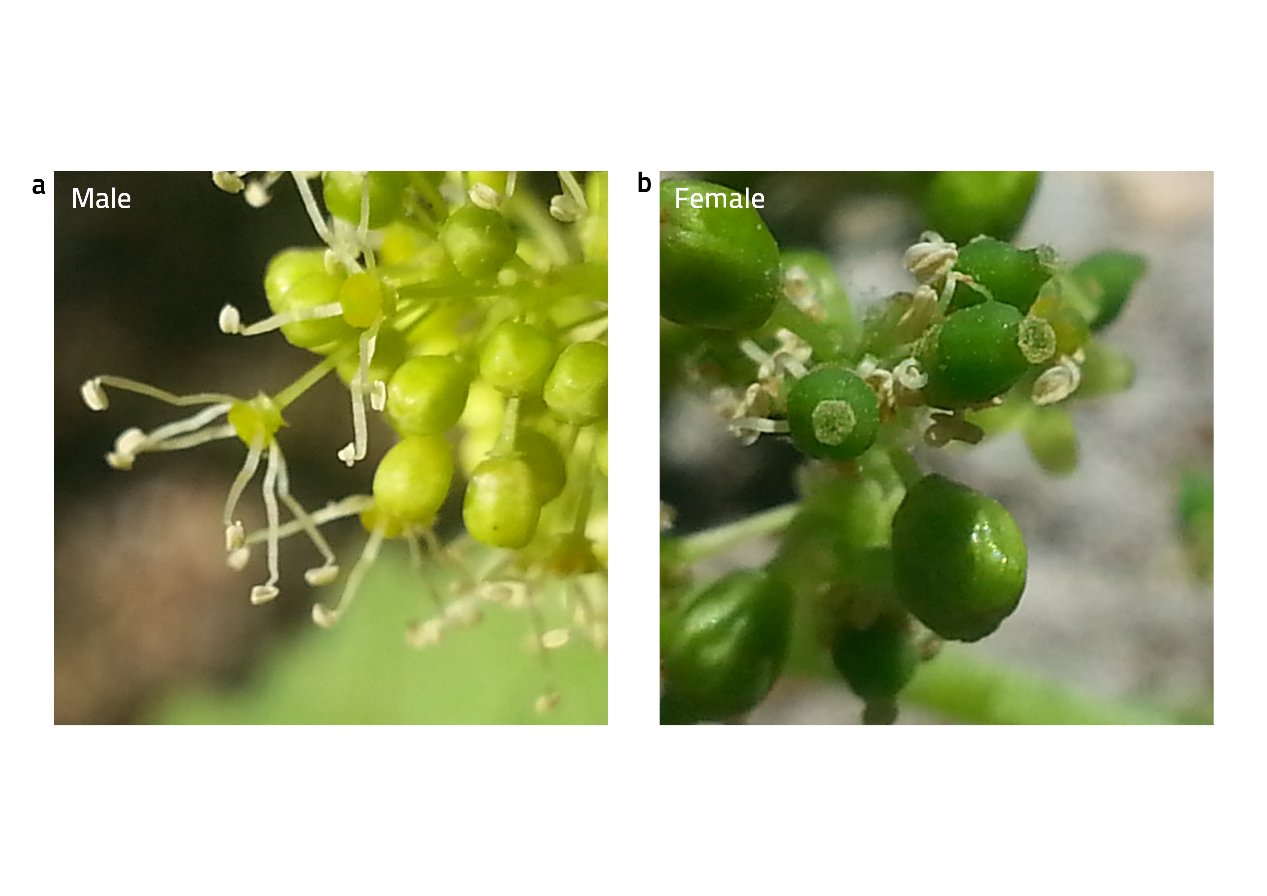


**Fig. S1: Male (a) and female (b) flowers of *Vitis vinifera* L. subsp. *sylvestris*.** The male flower contains only stamens, while the female contains a full carpel, and deteriorated stamens.

**
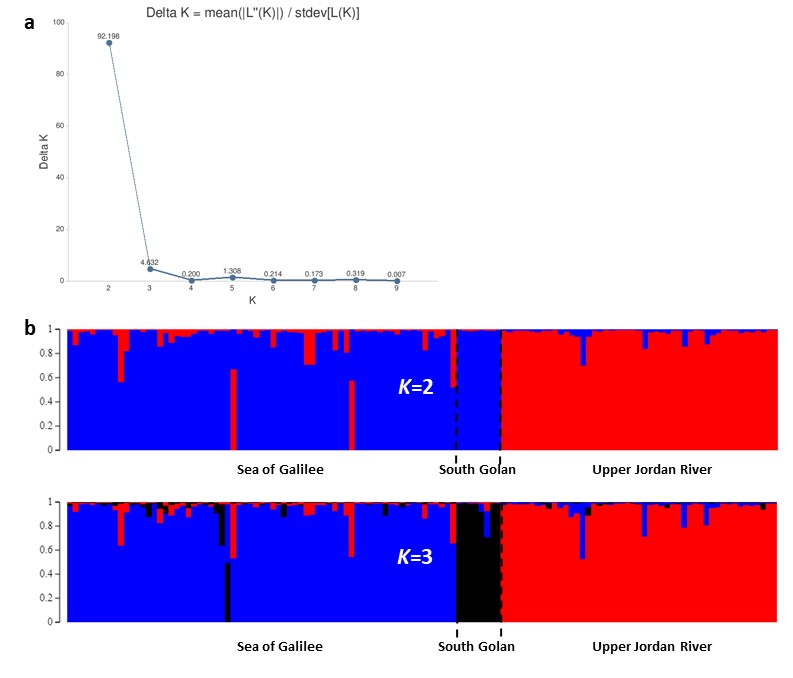
**

**Fig. S2:**  **Structure analysis of the population. (a)** Delta *K* graph based on twenty independent runs for each *K*, ranging from 1 to 9, were performed with a burn-in length of 5,000 followed by 50,000 iterations. The delta *K* was calculated based on the method proposed by Evanno, using CLUMPAK software. **(b)** Bar plots analysis for the K =2 and for the K=3. Each vertical bar represents a single individual accession.
